# Corticosteroid-Binding Globulin is expressed in the adrenal gland and its absence impairs corticosterone synthesis and secretion in a sex-dependent manner

**DOI:** 10.1038/s41598-019-50355-1

**Published:** 2019-09-30

**Authors:** José Gulfo, Ricard Castel, Angelo Ledda, María del Mar Romero, Montserrat Esteve, Mar Grasa

**Affiliations:** 1 Department of Biochemistry and Molecular Biomedicine, Faculty of Biology, University of Barcelona, Barcelona, Spain; 2 Institute of Biomedicine of the University of Barcelona, Barcelona, Spain; 3 CIBER Obesity and Nutrition, Institute of Health Carlos III, Madrid, Spain

**Keywords:** Adrenal cortex hormones, Adrenal gland diseases

## Abstract

Corticosteroid-binding globulin (CBG) is synthesized by the liver and secreted into the bloodstream where binds to glucocorticoids. Thus CBG has the role of glucocorticoid transport and free hormone control. In addition, CBG has been detected in some extrahepatic tissues without a known role. CBG-deficient mice show decreased total corticosterone levels with missing of classical sexual dimorphism, increased free corticosterone, higher adrenal gland size and altered HPA axis response to stress. Our aim was to ascertain whether CBG deficiency could affect the endocrine synthetic activity of adrenal gland and if the adrenal gland produces CBG. We determined the expression in adrenal gland of proteins involved in the cholesterol uptake and its transport to mitochondria and the main enzymes involved in the corticosterone, aldosterone and catecholamine synthesis. The results showed that CBG is synthesized in the adrenal gland. CBG-deficiency reduced the expression of ACTH receptor, SRB1 and the main genes involved in the adrenal hormones synthesis, stronger in females resulting in the loss of sexual dimorphism in corticosteroid adrenal synthesis, despite corticosterone content in adrenal glands from CBG-deficient females was similar to wildtype ones. In conclusion, these results point to an unexplored and relevant role of CBG in the adrenal gland functionality related to corticosterone production and release.

## Introduction

Glucocorticoids are steroid hormones secreted by the adrenal gland that perform pleiotropic functions. Their synthesis is regulated through hypothalamic-pituitary-adrenal axis (HPA)^1^ activity. Disturbance of the HPA axis leads to important physiological consequences that significantly affect energy metabolism and immune function among others^2^. Corticosteroid-binding globulin (CBG) is the main protein that carries glucocorticoids in blood and has high binding affinity for glucocorticoids. Thus, 80% to 90% of glucocorticoids in the bloodstream bind to CBG, a small proportion (5% to 10%) binds non-specifically to albumin and about 5% circulates freely^3^. According to the free hormone hypothesis^4^, the free fraction is available to interact with target cells and therefore act as the physiologically active hormone. In this context, CBG has the roles of transport glucocorticoids and control the amount of free hormone^5^. CBG is primarily synthesized by the liver and secreted into the bloodstream, where it binds to glucocorticoids. Oestrogens are potent inducers of the hepatic synthesis of CBG^6^, whereas interleukin-6 (IL6), insulin and glucocorticoids inhibit its expression^7,8^. In addition, CBG has been detected inside the cells of extrahepatic tissues such as white adipose tissue, lungs, heart, placenta and CNS^9–12^, although its role remains unknown. The enzyme elastase, which is secreted by activated neutrophils, has the capacity to cleave CBG, which consequently loses its affinity for glucocorticoids. This has been interpreted as a way to deliver glucocorticoids to the inflammation site^13^. A more active role for the modulation of glucocorticoid action by CBG is currently being explored^14^.

CBG-deficient mice show lower total corticosterone levels and higher free corticosterone and ACTH^15^, and greater adrenal gland size^16^, i.e., they have an altered response to the HPA axis. In addition, the sexual dimorphism that elicits increased levels of total corticosterone in females with respect to males is eliminated by CBG deficiency^17,18^.

The present study aimed to ascertain whether CBG deficiency affects the synthetic endocrine activity of adrenal gland in male and female mice. For this purpose, we investigated in adrenal glands from WT and CBG-deficient (KO) mice the expression of: the ACTH receptor (MC2R), the proteins involved in cholesterol uptake (scavenger receptor class B member 1, SRB1), and its transport to mitochondria where steroidogenesis takes place (steroidogenic acute regulatory protein, StAR) and the main enzymes involved in corticosterone, aldosterone and catecholamine synthesis. In addition, we also investigated whether the adrenal gland produces CBG. The results showed that CBG is present in the adrenal gland and its absence elicits a female-specific reduced expression of the main proteins involved in the adrenal hormone production resulting in the loss of the adrenal sexual dimorphism.

## Results

Table 1 shows the weight of the animals and their adrenal glands, in addition to total serum and adrenal corticosterone levels. As expected in females wild-type (FWT), the adrenal glands were heavier than in males wild-type (MWT) and corticosterone serum levels presented a corresponding increase in females. As previously shown, the serum corticosterone levels in KO mice were similar in males and females, and lower than those observed in WT mice^17^. By contrast, the weight of the adrenal glands in CBG-deficient (KO) mice was higher than that of WT mice and higher in females CBG-deficient (FKO) respect of males CBG-deficient (MKO) despite the lack of serum corticosterone differences. Corticosterone content in all the adrenal gland show dimorphism sexual in both WT and KO mice. Respect to WT, KO males showed similar corticosterone values in their adrenal glands whereas in females, KO accumulated corticosterone above WT levels without reaching significativity by Bonferroni post-test.

**Table 1:** Body, adrenal gland weights and serum and adrenal corticosterone levels in 12-weeks old female and male KO and WT mice.

Parameter	Unit	MWT	MKO	FWT	FKO
*Body weight*	*g*	34 ± 1	32 ± 1	24 ± 1^χ^	23 ± 1^χ^
*Adrenal weight*	*mg*	3.56 ± 0.16	4.90 ± 0.37*	5.40 ± 0.35^χ^	6.43 ± 0.46^χ^
*Adrenal weight*	%	0.011 ± 0.001	0.015 ± 0.001	0.023 ± 0.001^χ^	0.029 ± 0.002^χ^*
*Total corticosterone in serum*	*nM*	299 ± 48	179 ± 45	878 ± 99^χ^	266 ± 79*
*Corticosterone content in adrenal gland*	*pmol*	171 ± 51	147 ± 16	260 ± 63	465 ± 109^χ^

The significant results of two-way ANOVA were for body weight due to sex, p < 0.0001; adrenal weight in mg due to sex, p < 0.0001 and genotype, p = 0.0034; adrenal weight in % due to sex p < 0.0001 and genotype p = 0.0009; total corticosterone levels due to sex p > 0.0001, genotype, p > 0.0001 and interaction, p = 0.0003 and for corticosterone content in adrenal gland due to sex p = 0.0020 and interaction p = 0.0572. Significant Bonferroni post-test: *denotes a significant difference between WT and KO under the appointed sex and ^χ^denotes significant difference between males and females of the appointed genotype. Data are mean ± SEM of 6 to 10 mice.

**Figure 1:**
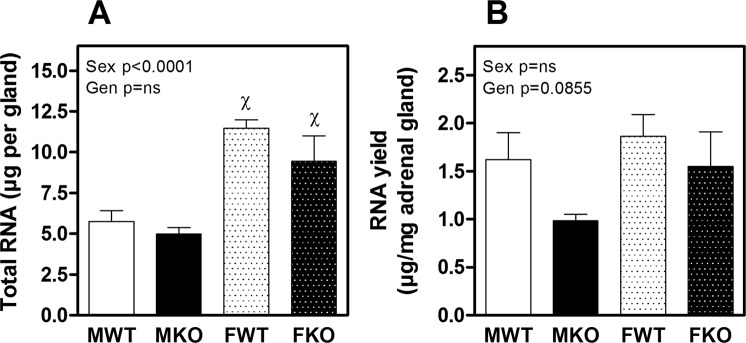
Total RNA (**A**) and RNA yield per mg of tissue (**B**) obtained from adrenal glands of males and females WT and KO (MWT, MKO, FWT and FKO respectively). The results of two-way ANOVA are specified in the graphs. Genotype (Gen) and sex (Sex) were the two variables assessed. Significant Bonferroni post-test: ^χ^denotes significant difference between males and females of the appointed genotype. Data are mean ± SEM of 6 to 10 mice.

The total mRNA obtained from adrenal glands was significantly higher in females than in males (Fig. 1A) because of their larger size (Fig. 1B), and there were no differences due to genotype (WT vs KO). The RNA yield in µg per mg of adrenal gland tended to be lower in KO mice of both genders, but there were no significant differences (Fig. 1B).

**Figure 2:**
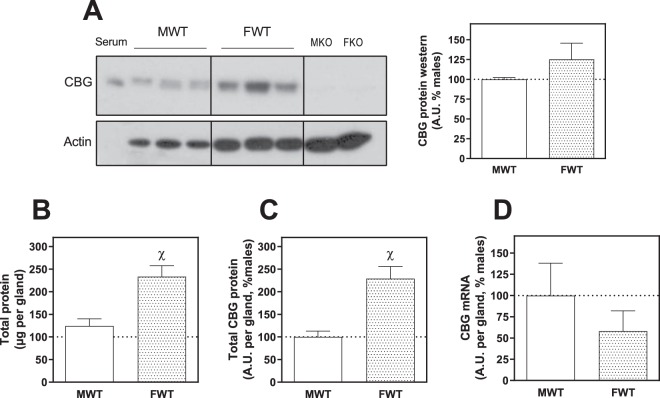
Western blot of CBG (**A**) in adrenal protein samples from males and females wild-type with serum as positive control and adrenal samples from KO mice as negative controls. Picture is cropped in order to eliminate samples of rats from experimental groups not included in this publication. The full picture is provided in the Supplementary file; **(B**) total protein per adrenal gland, (**C**) total CBG protein per whole adrenal gland and D) total mRNA of CBG per adrenal gland expressed as percentage of the expression in males WT. A.U.: arbitrary units. The results of t-student: ^χ^denotes significant difference between males and females WT. Data are mean ± SEM of 6 to 10 mice.

**Figure 3:**
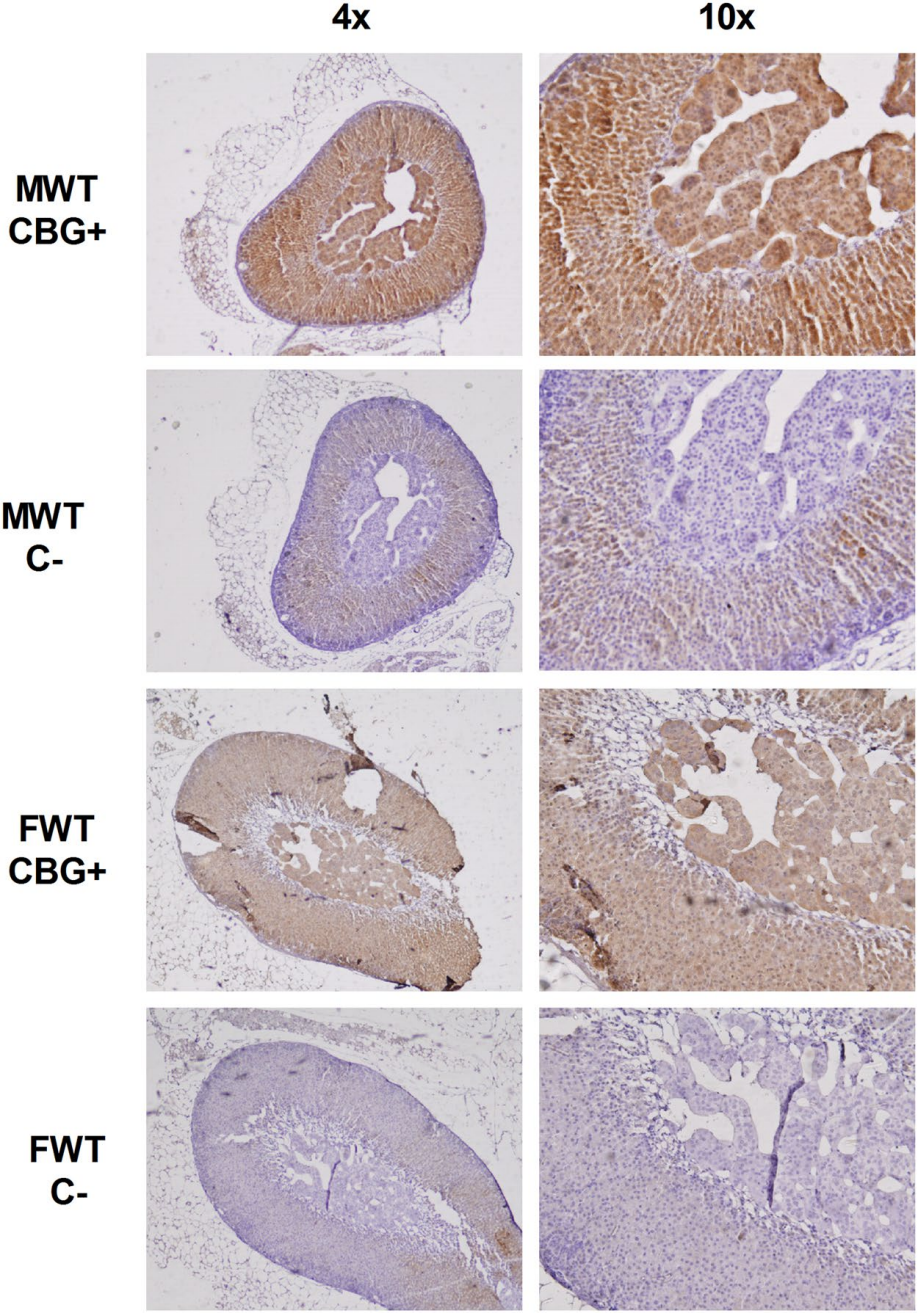
Representative CBG immunohistochemistry of perfunded adrenal glands from wild-type male and female mice. CBG + slides were incubated with anti-mouse CBG primary antibody. C- slides were negative controls without primary antibody incubation.

CBG mRNA and protein were found in the adrenal glands of WT males and females (Figs 2 and 3). The total protein (Fig. 2B) and total CBG protein (Fig. 2C) were higher in females than in males when expressed per gland, but these differences disappeared when CBG was calculated per µg of protein (Fig. 2A). However, total mRNA per gland tends to decrease in females without being significant (Fig. 2D).

The immunohistochemical analysis (Fig. 3) revealed a positive CBG signal inside the cells of the zona glomerulosa and the zona fasciculata, where glucocorticoids are synthesized. Nevertheless there was also a positive staining for CBG in the medullary zone, where the adrenal gland produces catecholamines, either in males and females.

**Figure 4:**
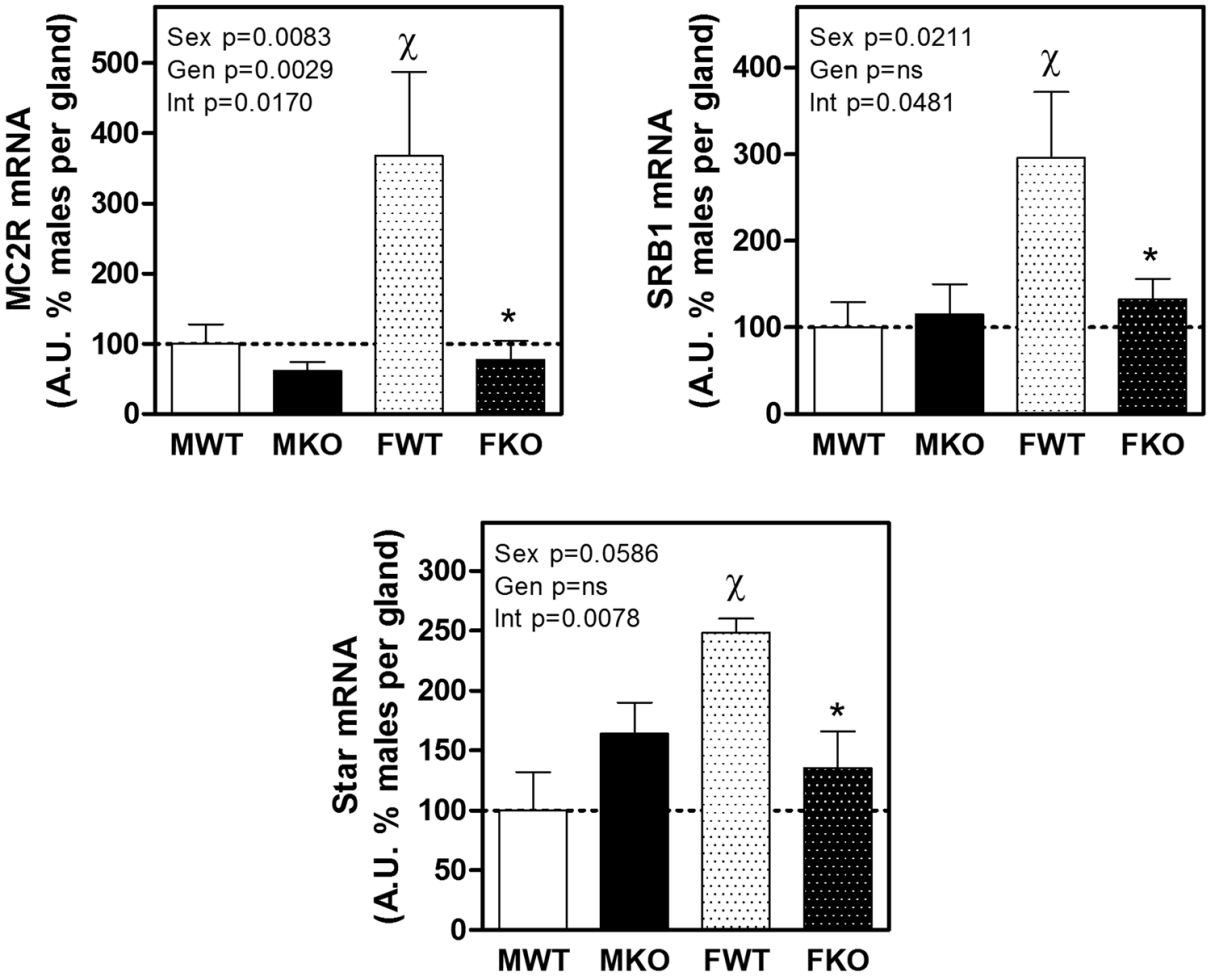
Expression of the ACTH receptor (MCR2), scavenger-receptor B1 (SRB1) involved in cholesterol uptake and StAR, the cholesterol carrier into the mitochondria. The results of two-way ANOVA are specified in the graphs. Genotype (Gen) and sex (Sex) were the two variables assessed. A.U.: arbitrary units. Significant Bonferroni post-test: *denotes a significant difference between WT and KO under the appointed sex and ^χ^denotes significant difference between males and females of the appointed genotype. Data are mean ± SEM of 6 to 10 mice.

Figure 4 shows the expression of the ACTH receptor MC2R, the main cellular cholesterol uptake receptor SRB1 and the protein that regulates cholesterol transfer within the mitochondria, StAR. According to the increased circulating corticosterone, females exhibited significantly higher expression of all three genes in WT mice. The KO mice displayed a reduced expression of MC2R mRNA, which was particularly evident in females. In the case of SRB1 and StAR, there were no significant differences between KO and WT males, but the expression of these two genes was greatly reduced in KO females.

**Figure 5:**
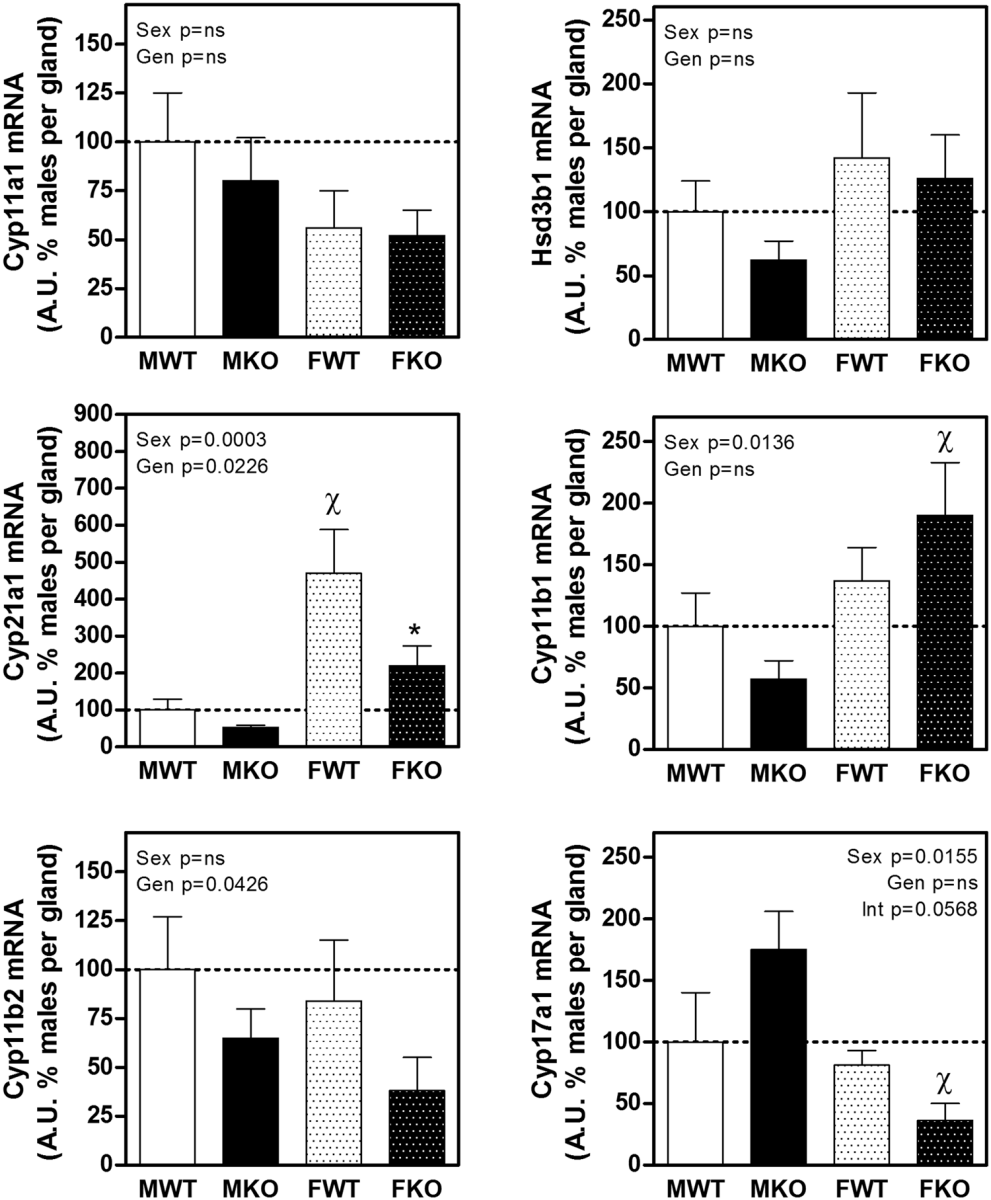
Expression of the main enzymes involved in steroid synthesis in the adrenal gland: Cyp11a1, Hsd3b1, Cyp21a1, Cyp11b1, Cyp11b2 and Cyp17a1. The results of two-way ANOVA are specified in the graphs. Genotype (Gen) and sex (Sex) were the two variables assessed. Interaction (Int) is included when p < 0.1. A.U.: arbitrary units. Significant Bonferroni post-test: *denotes a significant difference between WT and KO under the appointed sex and ^χ^denotes significant difference between males and females of the appointed genotype. Data are mean ± SEM of 6 to 10 mice.

The expression of the main enzymes involved in steroidogenesis is shown in Fig. 5. There were no differences between males and females or among WT and KO mice for Cyp11a1 or Hsd3b1. The expression of Cyp21a1, which catalyses the progesterone to deoxycorticosterone conversion, was much higher in females as compared to male WT mice. Deficiency of CBG diminished Cyp21a1 expression in both sexes of KO mice, and this effect was more pronounced in females.

Cyp11b1 expression was higher in females than in males KO, whereas Cyp17a1 displayed the opposite pattern, higher expression in males than in females KO mice. Of note, these sexual dimorphisms were not observed in WT mice. The expression of Cyp11b2 (corticosterone to aldosterone conversion) did not vary between WT and KO males and females, but CBG-deficient mice exhibited a reduced expression compared to WT mice in both sexes.

**Figure 6:**
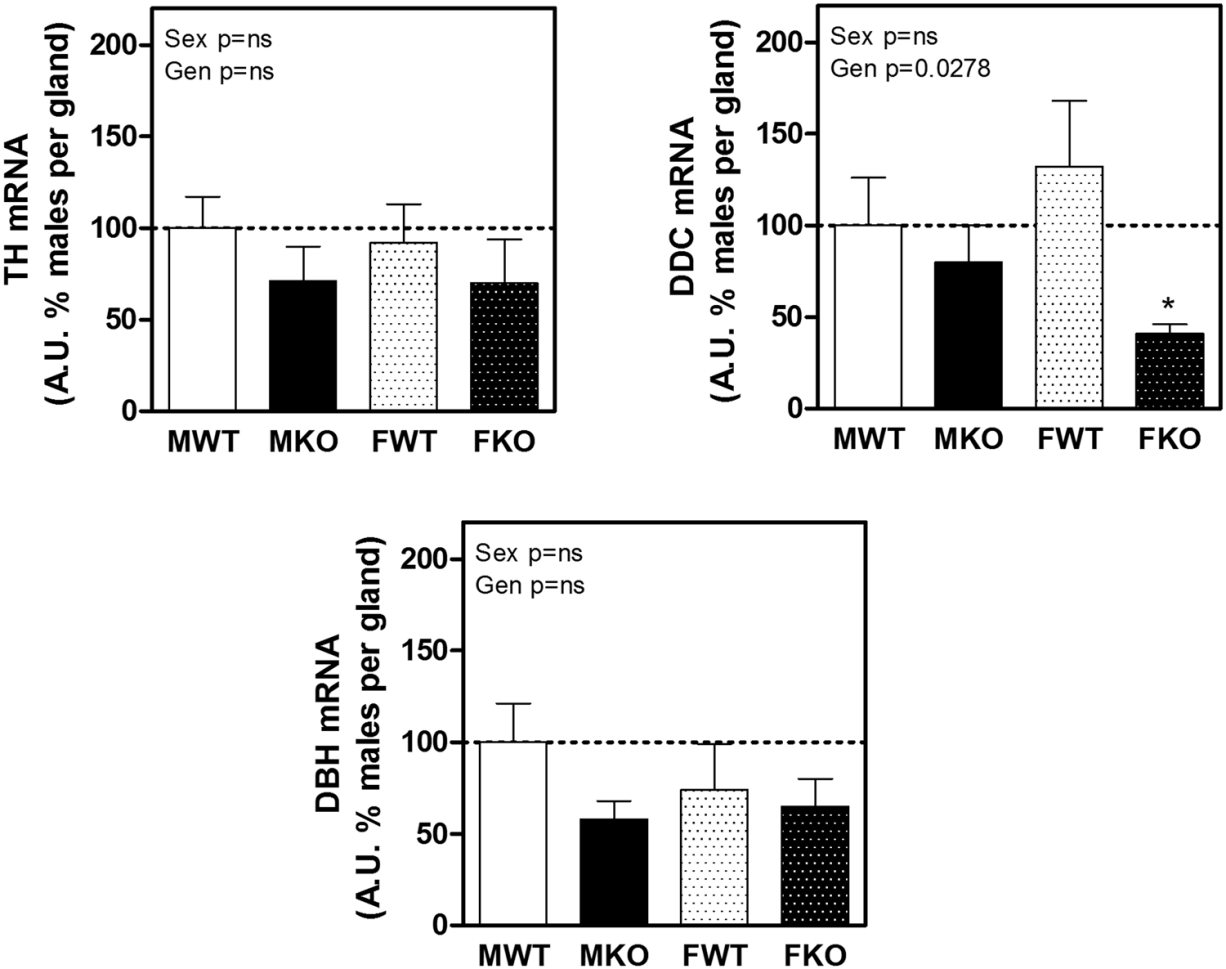
Expression of the main enzymes involved in catecholamine synthesis in adrenal gland: tyrosine hydroxylase (TH), dopamine β-hydroxylase (DBH) and DOPA decarboxylase (DDC). The results of two-way ANOVA are specified in the graphs. Genotype (Gen) and sex (Sex) were the two variables assessed. A.U.: arbitrary units. Significant Bonferroni post-test: *denotes a significant difference between WT and KO under the appointed sex and ^χ^denotes significant difference between males and females of the appointed genotype. Data are mean ± SEM of 6 to 10 mice.

Figure 6 shows the expression of enzymes involved in catecholamine synthesis. TH converts L-tyrosine to Dopa that in turn is transformed in dopamine by DCC. Noradrenaline is produced by DBH from Dopamine. Although the expressions tended to decrease in KO mice, the only significant difference was a reduction in DOPA decarboxylase (DDC) mainly in female KO, that catalyses the conversion of L-DOPA to dopamine.

## Discussion

In humans, it is well known that there are CBG polymorphisms that decrease its affinity for cortisol or its plasma levels^19–22^. The most common symptoms in humans carrying CBG mutations are fatigue, chronic pain, hypotension and, in some cases, overweight or obesity^19^. An inadequate response of HPA to stress is found in both humans and mice lacking functional CBG^18,19,23^. Deficient CBG mice show reduced total circulating corticosterone and increased free corticosterone levels^15–17^ and exhibit a higher corticosterone turnover rate^15^ that is consistent with a larger adrenal size^16^. We therefore investigated whether the lack of CBG had direct consequences on the adrenal functionality of the cortical and medullary zones. Furthermore, we determined whether adrenal glands produce CBG, as previously described in adipose tissue and lung^10,16,17^.

Here we show for the first time to our knowledge that adrenal glands express CBG, as previously described in other tissues^9,10,16,17,24,25^. Adrenal glands from males and females presented the same amount of CBG when expressed per µg of protein, while the levels were higher in females when expressed per gland, given the larger adrenal gland size in females compared to males. The adrenal gland is considered one of the most irrigated tissues^26^, so the CBG found by western blot may have come from blood. Although we cannot totally rule this out, the presence of non-negligible CBG mRNA (about 27–28 cycles) and the location of CBG in the cytosolic compartment within cells revealed by the immunohistochemistry analysis indicate that the CBG detected was adrenal in origin. We ruled out blood contamination in the immunohistochemistry analysis due to the perfusion with paraformaldehyde prior to the adrenal dissection. Even though corticosterone synthesis takes place at adrenal cortex level, CBG was also found in the adrenal medulla. A possible explanation is that CBG plays a role in the glucocorticoid regulation exerted on catecholamine synthesis^27^. The HPA axis is known to be involved in the regulation of catecholamine synthesis in the sympathetic ganglia and adrenal medulla of rats and mice^28,29^, but in a different way. An increase in ACTH promotes the expression of TH and DBH in sympathetic ganglia, but has no effect on their expression in the adrenal medulla^30^, since the MC2R receptor is only present in the adrenal cortex^31^. However, cortisol favours the synthesis of adrenaline from noradrenaline^32,33^ in the adrenal medulla^27^, so the presence of CBG could be interpreted as an intracellular carrier able to dose the glucocorticoids that drive adrenaline synthesis. This unexpected finding requires further research to ascertain the impact of CBG on adrenal gland functionality.

**Figure 7:**
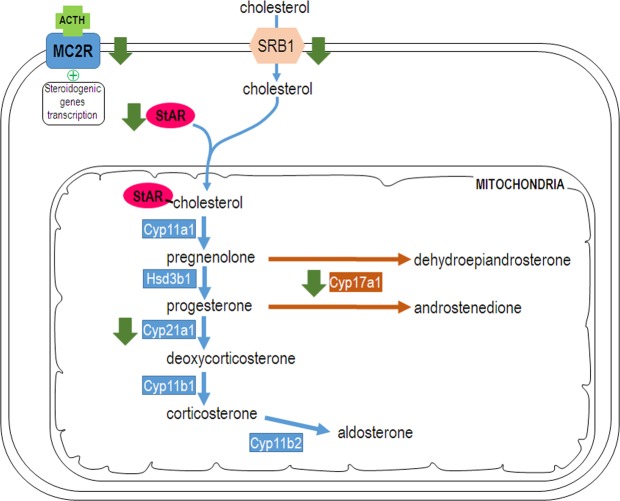
Schema of adrenal steroidogenesis where the enzymes that have been studied are shown. It is indicated with green down-arrows when the lack of CBG causes a decrease in their expression in females.

The expression of MC2R, the ACTH receptor, decreased in both male and female KO mice, but this effect was only significant in females. Previously, no altered response to ACTH had been reported from cultured adrenal cells of males CBG KO mice^34^, but there were no data from females that, according to our finding, resulted more affected. This contrasts with the high levels of ACTH previously described in KO mice^15^ and the upregulation exerted by ACTH on MC2R expression^35^ and points to a lower response capacity of the adrenal gland in CBG KO mice. The marked decrease in the uptake of cholesterol (SRB1) and its transport to the mitochondria (StAR) found in females are probably a consequence of the decreased response of the adrenal glands to ACTH that regulates both expressions^35,36^. The lack of differences between WT and KO males in StAR expression is in agreement with previous data^34^. The study on the expression of the enzymes involved in adrenal steroidogenesis revealed a decrease in KO mice of Cyp21a1, which directs progesterone to glucocorticoid synthesis^37^. Again, this effect was more pronounced in KO females in agreement with the reduced synthesis of glucocorticoids in the absence of CBG. On the other hand, Cyp11b1 expression in KO females showed a significant increase that could be a compensatory mechanism to the fall of substrate availability due to upstream enzyme down-regulation. Finally, the decrease in Cyp11b2 in male and female KO mice again points to a decrease in synthetic activity in the adrenal glands of KO mice (Fig. 7).

Ours results reinforce the observation that there is an adrenal weight increase in CBG-deficient mice^10^ and that a lack of CBG is associated with a loss of gender dimorphism in circulating corticosterone^17,18^. However, this lack of sexual dimorphism in circulating corticosterone has no translation in the corticosterone content found in adrenal glands. It is worth noting that, in female KO mice, the reduced expression of enzymes for corticosterone synthesis is accompanied by an enhanced corticosterone content. Higher adrenal size usually correlates with gland hyperactivity^38^, except in congenital adrenal hyperplasia (CAH)^39^. Patients with CAH present a natural mutation in one of the enzymes involved in cortisol synthesis, more frequently in Cyp21A2 referred also as 21-hydroxilase (classical CAH) analogous to Cyp21a1 in rodents, which leads to impaired cortisol and adrenalin production, larger adrenal size and less vulnerability to psychological stress^40^. Impaired cortisol production in CAH diminishes the negative feedback to the pituitary gland, that responds with enlarging the secretion ACTH, resulting in hyperplasia of the adrenal cortex^39^. According to this, CBG deficient mice with reduced circulating corticosterone levels also show increased of circulating ACTH levels^15^ resulting in adrenal hyperplasia^16^. Our data show that specifically female KO adrenals failed to secrete it, pointed by the increased corticosterone accumulation in the gland. Recently, it have been described that CBG plays a relevant role regulating the steroid hormones synthesis in testicular Leydig cells^24^. Overexpression of CBG promoted StAR and Cyp17a1 expression and progesterone release and CBG knockdown triggered the opposite response, decreasing enzyme expression and hormone release. These data has lead the authors to propose a new CBG role as hormone transporter from inside cell tissue. According to this role, in our model, the absence of CBG hinder the normal adrenal corticosterone production and release. The role of CBG is multiple and complex and its levels determine the HPA axis dimorphism in rats^41^. Here, we propose that CBG could facilitate corticosterone export from adrenal gland in stressful situations with high corticosterone output as fasting^42^. According to the double CBG action, females would be the more affected by CBG deficiency. Here we also found a decreased Cyp11b2 expression consistently with a mineralocorticoid synthesis decreases. Accordingly, individuals with CAH have mineralocorticoid synthesis decreased because the lack of substrate available for Cyp11b2 and resulting in a salt wasting^39^. Finally, the subjects with classical CAH with a block in cortisol biosynthesis shifts precursors to pathways that make excess adrenal-derived androgens^39^. Related to this, here we observed a tendency to increase, but not significant, the expression of Cyp17a1 in male KO and decreased expression in female KO mice. Given that this is poorly expressed in the adrenal glands of rodents^43^ (with a Ct of about 30 for Cyp17a1, while the rest of the enzymes presented a Ct of 15–20), the consequences in the overall androgen production are probably not relevant in CBG KO mice.

In conclusion, our results show that CBG is present in the adrenal gland and provide evidence that the lack of CBG leads to a reduction in the adrenal synthesis of glucocorticoids, and the capacity to secrete corticosterone in response to stressful stimuli^23,34^. The effect on adrenal glucocorticoid synthesis and secretion due to lack of CBG was strongest in females that could be related with different emotional reactivity in females previously reported^18^. The data here shown point to a relevant, yet unexplored, role for CBG enabling corticosterone synthesis and delivery to the blood and open the path to further studies unraveling this intriguing finding.

## Methods

### Animals and experimental protocol

Twelve-week old wild-type (WT) and CBG-deficient (KO) mice were used. The KO colony was established by crossing heterozygous breeding pairs, which were kindly provided by Dr. Willnow. The procedure to disrupt the CBG gene was described by Petersen *et al*.^15^. The mice were divided into four groups of six to 10 animals: WT males (MWT), KO males (MKO), WT females (FWT) and KO females (FKO). The animals were housed in a controlled environment, fed a standard laboratory pelleted formula (Teklad Global 2018, Harlan-Interfauna Ibérica, Sant Feliu de Codines, Spain) and had access to tap water *ad libitum*. Before killing, the mice were fasted overnight in order to induce corticosterone secretion. The mice were killed under isoflurane anaesthesia between 7 a.m. and 9 a.m., and the adrenal glands were carefully dissected, weighed and frozen at −80 °C until use. Blood samples were obtained and centrifuged to obtain serum. For the histological analysis, a number of animals, following their death, were perfused with paraformaldehyde to fix the tissue. All procedures were conducted in accordance with the guidelines for the use of experimental animals established by the European Union, Spain and Catalonia, and were approved by the University of Barcelona Animal Handling Ethics Committee.

### Total corticosterone

Corticosterone in serum and adrenals was measured by radioimmunoassay using a sheep anti-corticosterone antibody (AB1297, Millipore, USA) as described previously^44^.

### Western blot and corticosterone content from adrenal gland homogenates

Homogenates from one adrenal gland were prepared in 300 µL of PBS buffer containing 1/100 Complete™ protease inhibitor cocktail (Sigma). Samples were homogenized on ice using an Ultra-Turrax T10 (IKA). After a 1000 g centrifugation for 10 min at 4 °C, the pellet was discarded and the supernatant was collected and stored at −80 °C until use. Protein concentration of homogenates was measured with the Bradford reagent (Dye Reagent Concentrate, Bio-Rad Laboratories).

Adrenal homogenate samples (25 µg of protein) were separated by SDS/PAGE in a 10% acrylamide gel and electrotransferred to a PVDF membrane (Millipore). After blocking nonspecific binding sites with 5% nonfat milk and in TBSTween 0.05% for 90 minutes at room temperature, the membranes were incubated overnight with a CBG goat anti-mouse polyclonal antibody (LS-C39044, LifeSpan) and β-actin as control (A-5316, Sigma). The immunoreactive proteins were further detected by an anti-goat horseradish peroxidase-conjugated secondary antibody (sc-2922, Santa Cruz Biotechnology) and using Pierce ECL (Thermo Fisher Scientific). The quantification was carried out using Total Lab v2003.3 (Non-Linear Dynamics). Homogenate samples were deproteinized with cold acetone. After centrifugation, supernatants were dried under nitrogen gas and resuspendend 1/100 in PBS. Corticosterone content in these extracts was measured by radioimmunoassay as previously indicated.

### RNA isolation and RT-PCR

The total RNA from one adrenal gland was extracted using the TRI Reagent Solution (Ambion, Inc.). The RNA was quantified by measuring the absorbance at 260 nm and 280 nm using a NanoDrop ND-1000 spectrophotometer (NanoDrop Technologies). The cDNA was synthesized using MMLV reverse transcriptase (Promega) and oligo-dT primers (Attendbio), and reverse transcription was then performed using 2 μg of the RNA sample. Real-time PCR was conducted with SYBRGreen Master Mix (Life Technologies) on an ABI PRISM 7900 HT system (Applied Biosystems) using 10 μL of amplification mixtures containing 10 ng of reverse-transcribed RNA and 300 nM of the correspondent forward and reverse primers. Primer sequences used were: corticosteroid-binding globulin (CBG) forward: 5′-GGAAAATTGAGCATGTGGTCT-3′ reverse: 5′-AACTAATGTTGCCTGACTGG-3′; ACTH receptor (MCR2) forward: 5′-AGAACCAACATGAAGGGTGC-3′ reverse: 5′-AAGGGGGCCCAACAGAAGAT-3′; scavenger-receptor B1 (SRB1) forward: 5′-AAAGGGCTCCCAGGATAAGGA-3′ reverse: 5′-GAGTCCTCAAGAAGCGGGGT-3′; steroidogenic acute regulatory protein (StAR) forward: 5′-TGCCAACACCCACTCATACT-3′ reverse: 5′-CCTTCTTTGGGGTGTCTGCAT-3′; cytochrome P450 family 21a1 (Cyp11a1) forward: 5′-GGTTCCACTCCTCAAAGCCA-3′ reverse 5′-GGATCTCGACCCATGGCAAA-3′; 3beta-hydroxysteroid dehydrogenase isomerase 1 (Hsd3b1) forward: 5′-AAGCTGCAGACAAAGACCAAGG-3′ reverse: 5′-GCTTGAACACAGGCCTCCAA-3′; Cytochrome P450 family 21a1 (Cyp21a1) forward: 5′-CTTTCCTGCTTCACCACCCTG-3′ reverse: 5′-CCTTGGATGTTGGGGATGATG-3′; cytochrome P450 family 11b1 (Cyp11b1) forward: 5′-GCCTGAACGCTATATGCCTC-3′ reverse: 5′-CACGTGGAAGGATTTCAGCAC-3′; cytochrome P450 family 11b2 (Cyp11b2) forwad: 5′-AGCATCGCTGCAAATCCTCA-3′ reverse: 5′-GGTTTCGGCCCATGGAGTAG-3′; cytochrome P450 family 17a1 (Cyp17a1) forward: 5′-CTGGGCACTGCATCACGATA-3′ reverse: 5′-GATAAAGAGCTCCTGCCGGG-3′; tyrosine hydroxylase (Th) forward: 5′-CAGCCGGTGTACTTCGTGT-3′ reverse 5′-TCATCCTGGACCCCCTCTAAG-3′; dopa-decarboxylase (DDC) forward: 5′-TGCTACGCTTTGCTGTGTG-3′ reverse: 5-ACGAAGACGGAGTGGTAGTTA-3′; dopamine β-hydroxylase (DBH) forward: 5′-TGACTACGCCCCTATCTCCA-3′ reverse: 5′-GCGTGGAGGTGATCTTAGGC-3′ and for cyclophilin forward: 5′-GAGACTTCACCAGGGGAGATG-3′ reverse: 5′-GAGCCATTGGTGTCTTTGCC-3′. The specificity of the amplicons was determined by melting curve analysis. The ratio of the relative expression of target genes to cyclophilin was calculated by the ΔC(t) formula.

### Immunohistochemical analysis

Adrenals were obtained from 5 male and 5 female mice perfused with 40 g/L paraformaldehyde, embedded in paraffin and cut into 5 μm thick sections. For the immunohistochemical analysis, the sections were deparaffinized, rehydrated and washed in PBS-Tween. They were then treated with 0.3% hydrogen peroxide, blocked with 5% rabbit serum and incubated overnight at 4 °C with goat anti-mouse CBG (LS-C39044, LifeSpan BioSciences, USA) diluted 1:100. Then, slides were incubated with biotinylated rabbit anti-goat (1:400) and signal detection was performed with an avidin-biotin peroxidase complex (Vectastain ABC Kit, Vector Labs) and developed with diaminobenzidine hydrochloride chromogen (Sigma). The slides were visualized by light microscopy (Olympus BX-51) and image were captured with Olympus DP-70 Digital Camera System.

### Statistics

The data were expressed as means ± SEM and analysed using GraphPad 5.0. Statistical comparisons were obtained by two-way ANOVA to evaluate the impact of sex and genotype (named “Sex” and “Gen”, respectively, in the graphs). Interaction significance is also included (“Int”). A significant interaction would denote a different response in the mice of each genotype, depending on sex. The Bonferroni post-test was used as a multiple comparison method to evaluate significant differences between data pairs. For the CBG western results, the Student’s *t*-test was used to compare MWT and FWT mice because adrenals from KO mice, as expected, failed to show CBG. P values below 0.05 were considered significant.

## Supplementary information

Supplementary information

## Data Availability

All the data presented here will be available under request to the scientific community.
