# Supplementary material for: Corticosteroid-Binding Globulin is expressed in the adrenal gland and its absence impairs corticosterone synthesis and secretion in a sex-dependent manner
Source: Sci Rep. 2019 Sep 30;9:14018. doi: 10.1038/s41598-019-50355-1 (PMC6769001; doi:10.1038/s41598-019-50355-1)
Supplement: Supplementary file 1 — Supplementary information [file 41598_2019_50355_MOESM1_ESM.pdf]

Corticosteroid-Binding Globulin is expressed in the adrenal gland and its absence impairs corticosterone synthesis and secretion in a sex-dependent manner

José Gulfo[1,3], Ricard Castel[1], Angelo Ledda[1,3], María del Mar Romero[1,2,3], Montserrat Esteve[1,2,3,+], Mar Grasa[1,2,3,+,*]

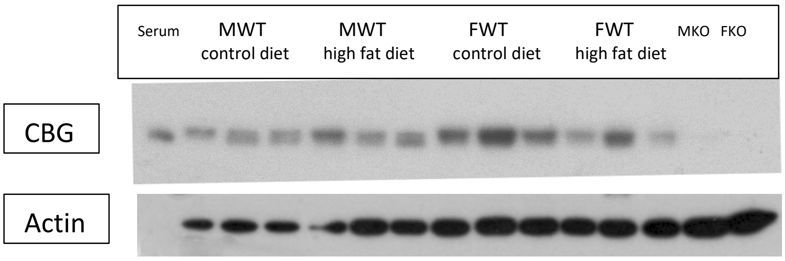

The original images from which we constructed figure 2 A (see it below) are shown. Some wells were excluded because the corresponding experimental groups (MWT and FWT fed with high-fat diet) are not treated in this research paper.

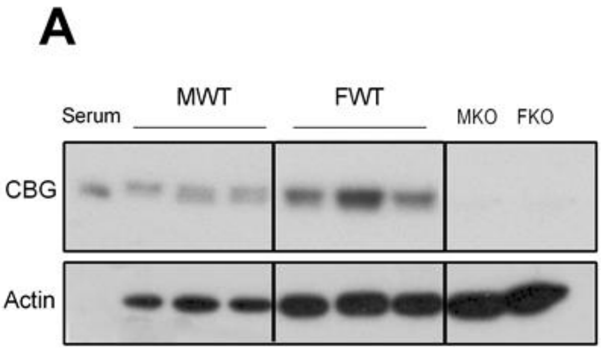

Figure 2. A) Western blot of CBG in adrenal protein samples from males and females wild-type with serum as positive control and adrenal samples from KO mice as negative controls.
